# Chikungunya Virus’ High Genomic Plasticity Enables Rapid Adaptation to Restrictive A549 Cells

**DOI:** 10.3390/v14020282

**Published:** 2022-01-28

**Authors:** Lien De Caluwé, Leo Heyndrickx, Sandra Coppens, Katleen Vereecken, Miguel E. Quiñones-Mateu, Andres Merits, Kevin K. Ariën, Koen Bartholomeeusen

**Affiliations:** 1 Virology Unit, Institute of Tropical Medicine Antwerp, 2000 Antwerp, Belgium; ldecaluwe@itg.be (L.D.C.); Lheyndrickx@itg.be (L.H.); scoppens@itg.be (S.C.); kvereecken@itg.be (K.V.); karien@itg.be (K.K.A.); 2 Department of Microbiology & Immunology, School of Biomedical Sciences, University of Otago, Dunedin 9054, New Zealand; miguel.quinones-mateu@otago.ac.nz; 3 Webster Centre for Infectious Diseases, University of Otago, Dunedin 9016, New Zealand; 4 Institute of Technology, University of Tartu, Nooruse 1, 50411 Tartu, Estonia; andres.merits@ut.ee; 5 University of Antwerp, 2000 Antwerp, Belgium

**Keywords:** alphavirus, Chikungunya virus, adaptation, trans-replicase assay

## Abstract

Chikungunya virus (CHIKV) is an emerging arthropod-borne virus that has spread globally during the last two decades. The virus is mainly transmitted by *Aedes aegypti* and *Aedes albopictus* mosquitos and is thus capable of replicating in both human and mosquito cells. CHIKV has a broad tropism in vivo, capable of replicating in various tissues and cell types but largely excluding blood cells. This was reflected in vitro by a broad array of adherent cell lines supporting CHIKV infection. One marked exception to this general rule is the resistance of the lung cancer-derived A549 cell line to CHIKV infection. We verified that A549 cells were restrictive to infection by multiple alphaviruses while being completely permissive to flavivirus infection. The adaptive growth of a primary CHIKV strain through multiple passages allowed the emergence of a CHIKV strain that productively infected A549 cells while causing overt cytopathic effects and without a fitness cost for replication in otherwise CHIKV-susceptible cells. Whole genome sequencing of polyclonal and monoclonal preparations of the adapted virus showed that a limited number of mutations consistently emerged in both structural (2 mutations in E2) and non-structural proteins (1 mutation in nsP1 and 1 mutation in nsP2). The introduction of the adaptive mutations, individually or in combinations, into a wild-type molecular clone of CHIKV allowed us to determine the relative contributions of the mutations to the new phenotype. We found that the mutations in the E2 envelope protein and non-structural proteins contributed significantly to the acquired phenotype. The nsP mutations were introduced in a split-genome trans-replicase assay to monitor their effect on viral genome replication efficiency. Interestingly, neither mutation supported increased viral genomic replication in either Vero or A549 cells.

## 1. Introduction

Chikungunya virus (CHIKV) is a single stranded RNA virus transmitted to humans through the bite of an infected *Aedes* mosquito, mainly *Aedes aegypti* and *Aedes albopictus* [1]. CHIKV was first described in 1952 in Tanzania [2], and the name, meaning “that which bends up”, is derived from the Makonde language [3]. CHIKV infection is characterized by a high fever, rash, and debilitating joint pains, which can persist for months to years, causing a significant burden on society [1,4,5]. Until 2004, *Aedes aegypti* was the primary vector of CHIKV, limiting its main distribution primarily to Africa and Asia. However, mutations in the envelope proteins (E1 and E2) have enabled CHIKV to use Aedes albopictus as an insect vector more efficiently. This allowed CHIKV, in combination with climate change and increased global travel and trade, to spread to previously unaffected regions (e.g., the Americas and Europe) [4,6,7]. The E1-A226V adaptive mutation to *Aedes albopictus* was first reported in 2005, allowing an increased midgut infection of *Aedes albopictus* but not of *Aedes aegypti* [8]. Since then, multiple other mutations of CHIKV related to *Aedes albopictus* adaptation have been identified in E2 (D60G, R198Q, L210Q, I211T, K233E, and K252Q [6,9,10,11]) and E1 (A98T [12]). Interestingly, the T98 residue in E1 found in all Asian strains completely blocks the E1-A226V adaptive mutation and thus prevents Asian strains from adapting to *Aedes albopictus* [12].

CHIKV belongs to the *Alphavirus* genus of the *Togaviridae* family. It is a small (60–70 nm) enveloped virus carrying a single-stranded positive sense RNA genome containing two open reading frames that respectively encode non-structural and structural proteins. The non-structural proteins nsP1, nsP2, nsP3, and nsP4 are directly translated from the genomic RNA and together form the replication complex. The replication complex is responsible for synthesizing the complementary negative-sense RNA viral genomes and 26S subgenomic (SG) RNAs. This SG mRNA is used to translate the structural proteins capsid, E3, E2, 6k, TF, and E1. Both non-structural and structural proteins are synthesized as precursor polyproteins that are proteolytically cleaved in individual proteins [1].

To complete its lifecycle, the virus replicates in the cells of both its mammalian and insect host. In humans, CHIKV is introduced in the skin by a mosquito bite and subsequently infects and replicates in various tissues and cell types in the skin, joints, liver, muscle, and the secondary lymphoid organs where fibroblast, epithelial cells, and macrophages were described to be infected [13,14]. In vitro, CHIKV similarly demonstrates a broad cell tropism and is capable of infecting a range of cell lines, although it is mainly limited to adherent cell lines and largely excludes blood cells [15,16,17,18]. Interestingly, one marked exception to the tropism of CHIKV for adherent cell lines is the lung cancer-derived A549 cell line, which is highly restrictive to CHIKV infection. A549 cells have been described as capable of supporting CHIKV binding, allowing translation of the non-structural proteins but restricting RNA replication [15,18], suggesting that CHIKV replication in A549 cells is hindered post-entry.

In this study, we applied viral passaging to adapt CHIKV to productive replication in restrictive A549 cells and subsequently characterized the adaptive mutations in the viral genome. The in vitro virus evolution system provides insights into the genetic plasticity of the virus, and the role of mutated viral proteins in overcoming cellular restrictions provides hints into the presence of cellular restriction factors as well. Interestingly, we found four non-synonymous amino acid substitutions, two in E2, one in nsP1, and one in nsP2, while one synonymous mutation was found in E1. The mutations in the ns-proteins were observed after only two passages, while the mutations in the structural proteins were identified in passage P7. The nsP1 mutation contributed most to the increased viral replication observed in both A549 and Vero cells. The adaptive mutations in E2 seemed specific to A549 cells and did not support increased viral replication in Vero cells, although viral attachment was not hampered. Using a trans-replicase assay, we could not identify an effect of the nsP mutations on the efficiency of the viral replication complex. Our results confirm the genomic plasticity of CHIKV that allows a rapid adaptation to cells, which are naturally restrictive to infection by this virus.

## 2. Materials and Methods

### 2.1. Cells and Viruses

A549 cells (ATCC CCL-185) were cultured at 37 °C and 7% CO_2_ in Dulbecco’s Modified Eagle medium (DMEM) supplemented with 10% fetal bovine serum (FBS) and 1% L-glutamine. Vero cells (ATCC CCL-81) were cultured at 37 °C and 7% CO_2_ in Eagle’s Minimal Essential Medium (EMEM) supplemented with 10% FBS and 1% L-glutamine.

The following viruses were used: a reporter CHIKV construct with a nanoluciferase gene inserted in nsP3 (Nanoluc CHIKV; ICRES1-P3Nanoluc [19]), CHIKV (strain: Indian-Ocean lineage (IOL) 06-049; pBR332-ChikFlic provided by M. Vignuzzi), Sindbis virus (SINV; EGAR 339, NCPV 0007163v), Eastern equine encephalitis virus (EEEV; H178/99, NCPV 0407041v) and Zika virus (ZIKV; MR 766, original stock provided by Bernhard-Nocht-Institut für Tropenmedizin, Hamburg). All virus stocks except the Nanoluc CHIKV were propagated on Vero cells and stored at −80 °C; Nanoluc CHIKV was produced in HEK293T (ATCC CRL-3216) cells.

### 2.2. Passaging of CHIKV

A549 cells were infected with the Indian-Ocean lineage (IOL) CHIKV strain. In a blind passage, the supernatant was transferred in a 1/1000 dilution to a new 6-well containing 1 × 10^6^ A549 cells. After every passage, aliquots of the supernatant were stored at −80 °C. Viral RNA present in the cell-free supernatant was quantified by RTqPCR using SYBR Green Master Mix (Biorad, Temse, Belgium) and primers E3F: AGTCTTGCCATCCCAGTTATGTGC; E3R: GCGTCGCTGGCGGTGGGGAG. The amount of viral envelope proteins present in the supernatant was determined by Western blot. As a primary antibody, anti-E2 (NR44002, Bei Resources, Manassas, VA, USA) was used, followed by incubation with the secondary anti-mouse antibody (ab6728, Abcam, Cambridge, UK).

### 2.3. Luciferase Assay

A549 and Vero cells were infected with a Nanoluc CHIKV. A serial dilution of the reporter virus stock was made in a culture medium containing 2% FBS. 24h after infection, nanoluciferase activity was measured using Nano-Glo^®^ Luciferase Assay System (Promega, Leiden, The Netherlands) on the TriStar LB 941 Multimode Microplate Reader (Berthold Technologies, Vilvoorde, Belgium).

### 2.4. Deep Sequence Analysis

Near full-length CHIKV genomes of the polyclonal or monoclonal viruses were sequenced using the MiSeq system (Illumina, San Diego, CA, USA). Briefly, viral RNA was purified (RNeasy Mini kit, Qiagen, Valencia, CA, USA), eluted in DNase/RNase-free water, and the viral genome RT-PCR amplified in two overlapping fragments using primers (Table 1). Dual indices (barcodes) and Illumina sequencing adapters were added by indexing PCR to the amplicons using Nextera XT DNA Library Preparation kit (Illumina), followed by DNA purification (Agencourt AMPure XP, Beckman Coulter, Brea, CA, USA). Individual barcoded DNA samples were then quantified (Qubit 2.0, Thermo Fisher Scientific, Mountain View, CA, USA), normalized to 4 nM, and pooled. Paired-end multiplexed libraries (up to 96 samples per deep sequencing run, including 5% PhiX as internal control) were diluted to 20 pM and denatured with NaOH prior to sequencing on the MiSeq system (Illumina) using the MiSeq Reagent Kit v3 600 cycle (2 × 300 bp, Illumina). Indexed reads were demultiplexed and filtered to remove short reads (<80 bp), generating sample-specific fastq files using BaseSpace (Illumina). Primary sequences from fastq files were aligned, and a phylogenetic tree was created with CLUSTAL W [20]. Contig sequences of the near-whole genome CHIKV deep sequencing data are available as Supplementary Material (Supplementary data S1).

### 2.5. Molecular Cloning

All mutants were introduced separately or together in pBR332-ChikFlic. Seven different constructs were made: E2-T196K, E2-H232R, E2-T196K-H232R, nsP1-M314V, nsP2-H687Y, nsP1-M314V + nsP2-H687Y and E2-T196K-H232R + nsP1-M314V + nsP2-H687Y using primers nsP1-M314V-F: GTGTGCAAGACTACCGACACGGTTG; nsP1-M314V-R: CAGGAATCCGTCTGCGTGGTGGG, nsP2-H687Y-F: TACACACCTTTTCGCATACACCATTACC, nsP2-H687Y-R: GATGTTTATGACCACTAGGTCATACC, E2-T196K-F: AGGTGCGGTACAAGTGTAATTGCGG, E2-T196K-R: TCTGGCCATTGACTGTGATCTTTACGTTGC, E2-H232R-F: GCAAAAAGTGGCAGTATAACTCCCC, E2-H232R-R: GATTGGTGACCGCGGCATGACATTG. Clones were verified by Sanger sequencing. Viral RNA was produced using the SP6 mMessage mMachine transcription kit (Invitrogen, Merelbeke, Belgium) following the manufacturer’s instructions. Viral RNA was transfected in Vero cells, and after 48 h, the viral stocks were harvested, filtered (0.45 µm), and stored at −80 °C. Viral stocks were titrated on Vero and A549 cells using a plaque assay, and TCID50 was calculated using the Spearman-Kärber method [21]. Stocks were quantified by RTqPCR, and TCID50 was normalized for input vRNA.

### 2.6. Trans-Replicase Assay (TRA)

The trans-replicase assay as described before [22] was utilized in an adapted version in this study. In brief, 30,000 A549 or Vero cells were plated in a 96 well plate one day before transfection. The template construct pU57-CHIKV-temp (2500 ng) was co-transfected with the replicase constructs (450 ng) containing nsP1-M314V, nsP2-H687Y, or both M314V and H687Y. A wild-type (WT) construct and a construct with defective polymerase activity (GAA) were used as controls. Transfections were performed using Fugene 6 (Promega, Leiden The Netherlands). After 17 h and 27 h, luciferase activities were measured using the Dual-Luciferase^®^ Reporter Assay System (Promega). Mutant TRA expressing plasmids were produced by TRA replicase plasmid digestion with EcoRV and PCR fragments carrying the nsP1 and nsP2 mutations insertion after PCR amplification from the corresponding ChikFLic molecular clone using primers nsP1-TRA-M314V-F: GTGTGCAAGACAACCGATACCG, nsp1-TRA-M314V-R: CAGAAAGCCGTCGGCGTGGTGGG, nsP2-TRA-H687Y-F: TACACCCCTTTCCGCATCCACCAC, nsP2TRAH687Y-R: GATGTTGATCACGACCAGGTCG. Clones were verified by Sanger sequencing.

### 2.7. Plaque Assay

80,000 A549 cells or 200,000 Vero cells were plated in a 24 well plate one day before infection. Cells were washed once with PBS and virus dilutions prepared in cell culture medium containing 2% FBS was added, and mixtures were incubated at 37 °C and 7% CO_2_ for 90 to 120 min. The virus dilutions were removed and the cells were washed two times with PBS. One ml of medium containing 10% FBS and 1% carboxy-methyl-cellulose (CMC) high viscosity was added, and the cells were incubated at 37 °C and 7% CO_2_ for 6 days. Subsequently, cells were washed two times with PBS and fixed with 1 mL of 3.7% formaldehyde for 30 min. Finally, cells were stained with 1 mL of naphthalene black solution for 30 to 60 min, after which the wells were washed with water.

To prepare plaque-purified monoclonal viruses, 600,000 A549 cells were seeded in a 6 well plate one day before infection. Cells were washed once with PBS, virus dilutions prepared in cell culture medium containing 2% FBS were added, and mixtures were incubated at 37 °C and 7% CO_2_ for 2 hr. The virus was removed and the cells were washed twice with PBS. Three ml of a 0.3% agarose overlay in DMEM was added, and the cells were incubated at 37 °C and 7% CO_2_ for 6 days. Subsequently, 1.5 mL of a 0.5% agarose overlay containing Neutral Red was added and incubated for another 20 min. A pipette tip was inserted into the agar overlay to pick up a single plaque. The tip was immersed in cell culture media, and the clonal virus was expanded on A549 cells.

### 2.8. Binding Assay

100,000 A549 or Vero cells were plated in a 24 well plate. The next day, a cold medium was added to the cells for 30 min at 4 °C. Cells were incubated with 10-fold dilutions of WT CHIKV or equal amounts of adapted CHIKV for 90 min at 4 °C. Stocks of WT and adapted CHIKV were quantified by RTqPCR. Cells were washed four times in a cold medium with 2% FBS. After washing, the cells were re-suspended in TRIzol. RNA was purified, and viral genomes were quantified using RT-qPCR using SYBR Green Master Mix (Biorad, Temse, Belgium) and primers E3F: AGTCTTGCCATCCCAG-TTATGTGC; E3R: GCGTCGCTGGCGGTGGGGAG. A standard curve was produced from in vitro transcribed CHIKV RNA to calculate CHIKV vRNA copies.

## 3. Results

### 3.1. CHIKV Replication Is Restricted in A549 Cells

**Figure 1:**
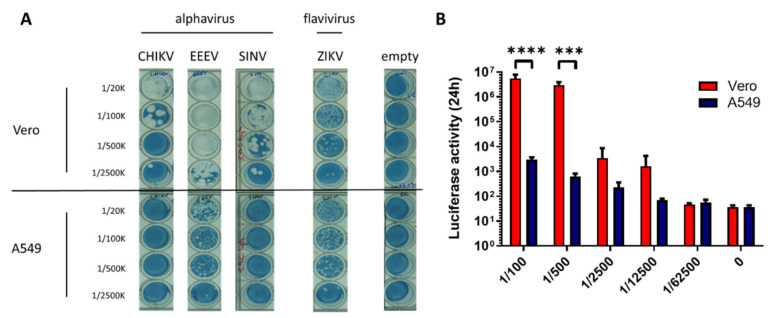
Infectivity of CHIKV on Vero and A549 cells. (**A**) Plaque assay of CHIKV, SINV, EEEV, and ZIKV on Vero and A549 cells. (**B**) Vero and A549 cells were infected for 24 h with a serial dilution of Nanoluc CHIKV, whereafter luciferase activity was measured. Mean ± SD is shown (*n* = 3). Two-way ANOVA with Šidák multiple comparisons was performed *** = *p* < 0.001 and **** = *p* < 0.0001.

To verify the restriction of CHIKV replication in A549 cells, the infectivity of CHIKV and related alphaviruses was assessed on both A549 and Vero cells using both a plaque assay and a nanoluciferase reporter virus. For the plaque assay, a clinical isolate of CHIKV (IOL 06-049) was used. We demonstrated that A549 cells were restrictive for CHIKV (Figure 1A). Also, another Old World alphavirus, Sindbis virus (SINV), was not capable of productively infecting A549 cells, evidenced by a lack of plaques (Figure 1A). For the New World alphavirus Eastern equine encephalitis virus (EEEV), A549 cells were permissive, although a smaller plaque phenotype was observed compared to Vero cells (Figure 1A), suggesting restricted viral replication. No difference in permissiveness was observed for flavivirus Zika virus (ZIKV) between A549 and Vero cells (Figure 1A). Since the read-out of a plaque assay is based on the cytopathic effect, no differentiation between a defective viral replication or a lack of cytopathy could be made. In order to assess the replication capacity of CHIKV in A549 and Vero cells, a reporter CHIKV virus carrying a nanoluciferase reporter gene in nsP3 (Nanoluc CHIKV) was used to infect both A549 and Vero cells. An 1881-fold and a 4772-fold lower nanoluciferase activity were observed in A549 cells compared to Vero cells in the two lowest dilutions (Figure 1B). This indicates that the absence of plaques is not merely due to the absence of cytopathy but to a replication block present in A549 cells. Altogether, we confirm the restriction of CHIKV replication in A549 cells and extend this to related alphaviruses, while A549 cells are completely permissive to a prototype flavivirus infection.

### 3.2. Adaptation of CHIKV on A549 Cells

**Figure 2:**
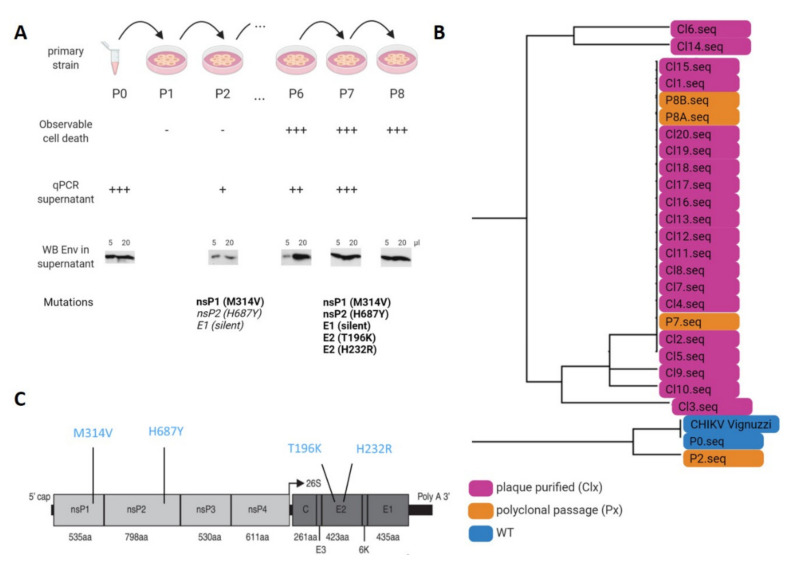
Serial passaging of CHIKV on A549 cells. (**A**) Serial passaging strategy used for adaptation of CHIKV (IOL) to A549 cells. The amount of cell death was monitored during this protocol. Viral RNA in cell-culture media was measured by RT-qPCR after P0, P2, P6, and P7. Increased presence of RNA genomes or cell death is indicated with “-“, “+”, “++” and “+++”. Viral envelope proteins were determined by western blot by loading 5 or 20 µL of cell culture supernatant after P0, P2, P6, P7, and P8. Deep sequencing identified adaptive mutations in P0, P2, P7, and P8 stocks. Present mutations are indicated; mutations in italic are presented as a mixture. (**B**) Phylogenetic tree generated using Clustal W generated multiple sequence alignment of sequenced passages P0, P2, P7, and P8 and 20 plaque-purified monoclonal viruses from P8 (**C**) Location of mutations found in CHIKV after adaptation to A549 cells.

To gain insight into the determinants that govern the restriction of CHIKV replication by A549 cells, the IOL 06-049 CHIKV strain was serially passaged eight times. During the continuous passaging, the observed cell death gradually increased, coinciding with an increasing amount of viral RNA and viral envelope proteins in the supernatant (Figure 2A). To identify mutations potentially responsible for this adaptation, the near full-length CHIKV genome from passages P0, P2, P7, and P8 together with 20 individual plaque-purified monoclonal viruses derived from P8, were RT-PCR amplified in two overlapping fragments (Table 1) and deep sequenced (Supplementary Figures S1–S4). The originating virus before adaptation was designated as passage 0 (P0) and served as control. P2 was sequenced as it represented the first discernable breakthrough growth in A549 cells. P8 was expected to be a fully adapted virus as no further increase in infection potential could be noticed. To verify this, the earlier P7 and 20 monoclonal viruses derived from P8 were additionally sequenced. A phylogenetic tree was produced by multiple sequence alignment of the viral genomes (Figure 2B), showing separate clustering of the early passage genome (P2) with the originating virus (P0 and the CHIKV reference sequence). The later passages, P7 and P8, clustered separately from P0 and P2, and together with the 20 plaque-derived monoclonal virus genomes derived from P8 (Figure 2B). In the fully adapted P7 and P8 polyclonal and the plaque-purified monoclonal viruses, four non-synonymous mutations were consistently found, one in nsP1 (M314V), one in nsP2 (H687Y), and two in E2 (T196K and H232R) (Figure 2C and Supplementary Figures S1–S4). Additionally, one synonymous mutation was identified in E1. The mutations in E2 were not yet present in P2 and thus must have been acquired during later passages. The nsP1 mutation was already present in P2 and thus likely represents the earliest adaptation of CHIKV to A549 cells. The nsP2-H687Y mutation and the silent mutation presented a mixture of WT (H687) and mutant virus (Y687) in the P2 supernatant, suggesting that these mutations followed relatively quickly after the nsP1-M314V mutation. These observations show that only a limited number of mutations were required to adapt CHIKV to the restrictive A549 cell line and that this adaptive process occurred relatively fast.

### 3.3. Contribution of the Identified Mutations to the Viral Phenotype

To determine the relative contribution of the adaptive mutations on the acquired phenotype, the mutations were introduced in a CHIKV molecular clone as individual mutations and together. Seven different constructs harboring following mutation(s) were made: E2-T196K, E2-H232R, E2-T196K-H232R, nsP1-M314V, nsP2-H687Y, nsP1-M314V + nsP2-H687Y and E2-T196K-H232R + nsP1-M314V + nsP2-H687Y. Viruses were rescued, obtained viral stocks were titrated on both Vero and A549 cells using a plaque assay, and TCID50 was calculated and normalized for viral input RNA (vRNA).

**Figure 3:**
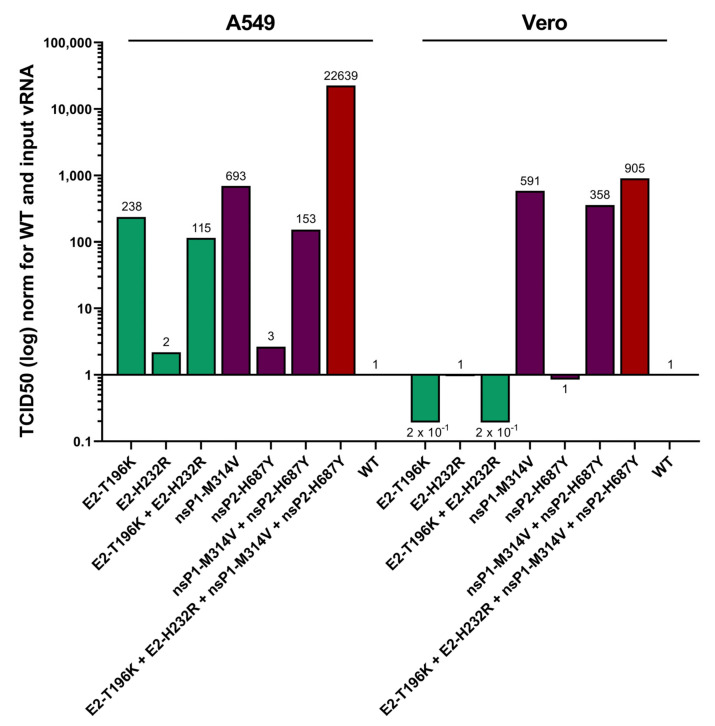
Effect of A549 acquired mutations on infectivity in Vero and A549 cells. Vero cells were transfected with viral RNA carrying the different mutations. After 48h, supernatant was harvested, and TCID50 on Vero and A549 cells was calculated. TCID50 was calculated using the Spearman-Kärber method from 6 replicates of each titration. TCID50 was first normalized for input vRNA determined using RTqPCR followed by normalization for WT CHIKV for each cell line.

In A549 cells, the viral envelope E2-T196K mutation contributed significantly to the increased viral replication of the adapted virus. The E2-T196K mutant stimulated viral replication 238-fold while a small or no additional effect of the E2-H232R mutation alone or in combination with E2-T196K, respectively, was observed (Figure 3). Interestingly, the nsP1-M314V mutation contributed most to the acquired phenotype with a 693-fold increase in viral replication in A549 cells. Only a negligible increase in viral replication was noticed when nsP2-H687Y was introduced separately. No additive effect was observed when nsP2-H687Y was introduced together with nsP1-M314V, leading instead to a slightly diminished 153-fold increase in CHIKV replication when introduced together. When all four mutations were combined, a synergistic effect became apparent, with a 22,639-fold increase in viral replication in A549 cells (Figure 3).

In contrast to the A549 cells, when introducing the envelope E2 mutants in Vero cells either separately or in combination, viral replication was impeded (Figure 3). After introducing the E2-T196K mutation, a 5-fold decrease in TCID50 on Vero cells was observed. No decrease or increase was seen after the introduction of the E2-H232R mutation. Introducing the two E2 mutations together led to a 5-fold decrease in viral replication in Vero cells. When introducing the mutations in the non-structural proteins that arise after adaptation in A549 cells, a similar increase in viral replication was seen in Vero cells. Similar to A549 cells, the introduction of the nsP2-H687Y mutant alone led to a small decrease in viral replication in Vero cells, while the introduction of the nsP1-M314V alone or together with nsP2-H687Y, respectively, increased viral infectivity of CHIKV 591-fold and 358-fold in Vero cells. Surprisingly, the introduction of the E2 mutants in addition to the nsP mutants led to a minimal further increase in CHIKV replication (905-fold) in Vero cells (Figure 3), suggesting reciprocal interactions between the mutations beyond simple additive effects.

In conclusion, the envelope localized E2-T196K-H232R mutations are required to adapt CHIKV to A549 cells but suppress replication in Vero cells. The nsP1-M314V mutation allows increased viral replication in both A549 and Vero cells. The nsP2-H687Y mutant has minimal effect on viral replication in both cell types. The four mutations acquired by adapting CHIKV to A549 cells not only allowed increased infectivity in A549 cells but also in Vero cells.

### 3.4. Mutations in nsP’s Have a Role Outside the Replicase Complex

**Figure 4:**
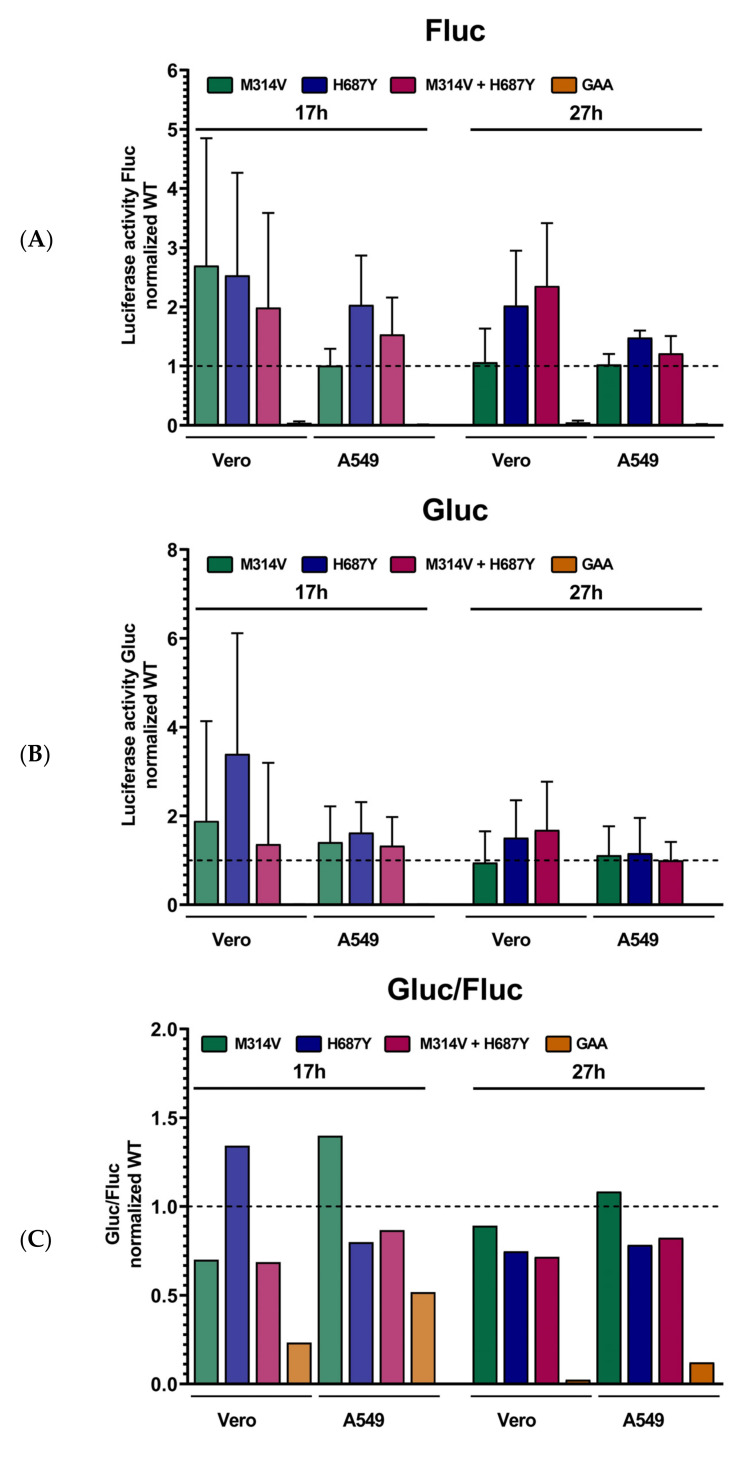
Trans-replicase assay to test role nsP mutations on viral replication complex efficiency. (**A**) Fluc activity, (**B**) Gluc activity and the (**C**) ratio Gluc/Fluc are shown. Activity was measured after 17 h and 27 h. nsP1 mutation M314V and nsP2 mutation H687Y were tested separately and combined. The nsP4 GAA catalytic mutant was used as a control as it abolishes viral polymerase activity. All data were normalized to a WT control, indicated by the dashed line, for each time point and cell line. At least two biological replicates (*n* = 4) were performed. Mean ± SD is shown.

A split-genome trans-replicase assay (TRA) [22] was used to investigate the role of the nsP mutations on viral RNA replicase activity. In the trans-replicase system, the viral RNA replication and the expression of the nsP proteins are uncoupled [22,23,24]. Plasmids coding for the viral replicase proteins is contransfected with the reporter construct containing all the trans-regulatory elements of the viral genome, in which the non-structural protein and the structural protein-coding sequence are replaced with ae *Firefly* luciferase (Fluc) and *Gaussia* luciferase (Gluc) reporter gene respectively. As such, the FLuc activity reflects the overall activity of the replication complex, and the Gluc expression, controlled by the subgenomic (SG) promotor, correlates with late-stage SG RNA synthesis. Replicase expressing plasmids carrying nsP1-M314V and nsP2-H687Y alone or together were created and tested alongside the WT and nsP4-GAA catalytic dead mutant as controls in both A549 and Vero cells. The different luciferase activities were measured at two time points, 17 h and 27 h (Figure 4). No clear differences in full-length RNA synthesis were observed between the constructs carrying the adaptive mutations, shown by their Fluc activity (Figure 4A). By contrast, the nsP4 GAA mutation which abolishes viral RNA polymerase activity resulted in much lower Fluc activity in all tested cell lines and at all time points (Figure 4A). As expected, Gluc activity in the cells co-transfected using nsP4 GAA mutant was almost absent (Figure 4B). The adaptive mutations did not definitively increase Gluc activity or alter the Gluc/Fluc ratio compared to their WT control (Figure 4B,C). After 17h, a suggested small increase in Gluc activity could be observed for the three different constructs carrying the adaptive mutations in both cell lines. However, at the next time point this effect disappeared (Figure 4B). Similarly, the Gluc/Fluc ratio of the nsP2-H687Y in Vero cells and the nsP1-M314V in A549 cells was slightly increased at the 17h time point. However, this elevation disappeared at the next time point (Figure 4C). Overall, no clear increased efficiency of the viral replication complex was observed (Figure 4B,C). While the nsP1-M314V mutation was critical for increased viral replication in the context of viral replication (Figure 3), no effect was observed in the TRA (Figure 4). Neither did the nsP2-H687Y mutation nor the combined nsP1-M314V and nsP2-H687Y mutations increase replicase complex efficiency. Altogether, this suggests a role for the adaptive nsP mutations separate from the catalytic function of the replicase complex.

### 3.5. Adaptive Mutations in the Envelope E2 Protein Enhance Attachment to Both A549 and Vero Cells

**Figure 5:**
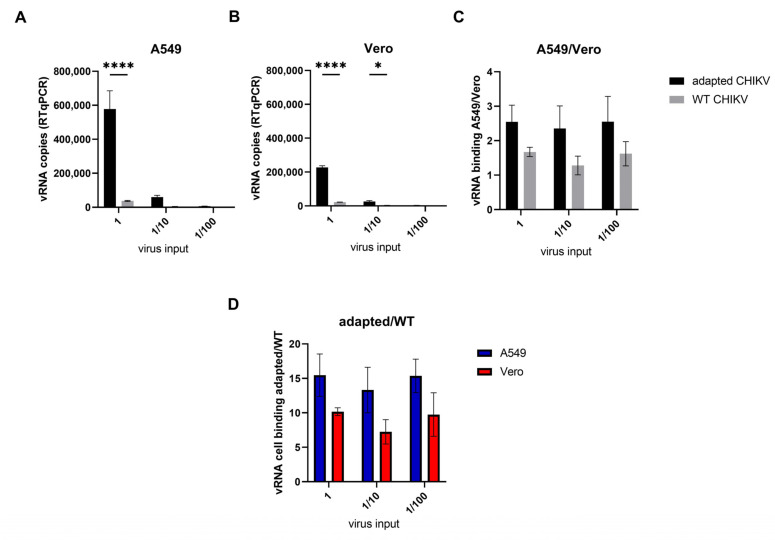
Binding assay to evaluate attachment of WT and E2 adapted virus carrying T196K and H232R mutations to A549 and Vero cells. (**A**) Binding of WT and adapted virus to A549 cells (**B**) Binding of WT and adapted virus to Vero cells (**C**) Ratio of virus binding A549/Vero cells is shown for WT and adapted virus. (**D**) Ratio virus binding adapted/WT is shown for both A549 and Vero cells. Three 10-fold dilutions of CHIKV were incubated with each cell line. Equal amounts of viral genomic RNA were used for the WT and adapted CHIKV. Mean ± SD is shown. Two-way ANOVA with Šidák multiple comparisons was performed * = *p* < 0.05 and **** = *p* < 0.0001.

While the adaptive mutations in the structural proteins positively impacted viral replication in A549 cells, the opposite was observed in Vero cells (Figure 3). Since the envelope proteins mediate viral attachment and binding, the role of these adaptive mutations on virus attachment was investigated using a cellular binding assay comparing the binding of WT virus and E2 adapted virus (E2-T196K-H232R) to both Vero and A549 cells. Equal amounts of WT or E2 adapted virus (1.32 × 10^7^ vRNA copies), in three 10-fold dilutions, were allowed to bind for 90 min at 4 °C to both A549 and Vero cells. After extensive washing, the cell-associated viral RNA was determined by RTqPCR (Figure 5A–D). Interestingly, the E2 adapted virus was found to associate better with both A549 and Vero cells compared to the WT virus (Figure 5A,B) independent of the amount of input virus. When we compare the relative binding of the viruses to A549 and Vero cells, a slightly increased, although not significant, attachment of the E2 adapted virus to A549 can be observed compared to Vero cells (Figure 5D). Similarly, when we compare the amount of attached WT and adapted virus to each cell line, a better attachment of the adapted virus to A549 than to Vero cells can be observed compared to the WT virus (Figure 5C). Overall, the adaptive T196K and H232R mutations in the CHIKV envelope proteins improve viral attachment to both A549 and Vero cells while they only confer increased infectivity in A549 cells. The relative increased binding to A549 cells compared to Vero cells by the virus carrying the T196K and H232R mutations in the viral envelope protein could indicate increased binding to an A549 membrane factor that allows increased entry of the virus.

## 4. Discussion

CHIKV has a broad tropism both in vivo and in vitro, as demonstrated by the wide range of susceptible primary cell types and cell lines [13,14,15,16,17,18]. In vitro CHIKV shows a preference for adherent cell lines as it only has limited support for CHIKV infection in suspension cells. The adherent cell line A549, however, is an exception to this rule since it is restrictive for CHIKV replication. These cells were confirmed to be resistant to infection by CHIKV and related alphaviruses, while these cells were partly permissive for EEEV and completely permissive to flavivirus infection. This agrees with the literature where CHIKV has previously been shown to be capable of binding to A549 cells while virus replication was impeded [15,18]. A549 cells are, however, susceptible to infection with lentiviral particles carrying the CHIKV envelope, and evidence of non-structural protein translation has been found, suggesting that CHIKV replication is restricted in A549 cells post-entry, but the block is not related to replicase proteins translation [15,16,17]. Here, we serially passaged a CHIKV strain on A549 cells and found that CHIKV only required four mutations to allow potent CHIKV replication in A549 cells. These adaptive mutations were rapidly selected under the applied in vitro conditions.

Resulting from adaptation to A549 cells, two mutations in the envelope E2 protein were unexpectedly identified. They positively contributed to the acquired phenotype while having a negative effect on CHIKV replication in Vero cells. In addition, the acquired mutations improved viral attachment to both A549 and Vero cells, suggesting that virus internalization is impacted differently in either cell line. These two mutations in E2 are located in close proximity in the folded protein (Supplementary Figure S5). In these E2-T196K and E2-H232R adaptive mutations, the Thr and His residues were substituted by a Lys or Arg residue. Reversal to the positively charged Lys or Arg residues is often observed in cell culture adapted CHIKV strains, and other alphaviruses where several point mutations of positively charged amino acids in E2 (e.g., E79K, G82R, and E166K) have been described [25,26,27,28]. These positively charged amino acids facilitate an increased engagement of glycosaminoglycan (GAG) molecules to tether the virus to the cell membrane, which could eventually lead to a more efficient entry of the viral particles [29]. Although such adaptations have previously been found, the ones we describe here have not been reported before in cell culture adapted or primary CHIKV strains. Since the adaptive mutations in E2 have opposite effects in A549 and Vero cells, which cannot be explained by a difference in attachment to these cell lines, this could suggest a different GAG production, presentation, or usage in these cell lines. Possibly the GAG-presenting factor that allows increased uptake in A549 cells is absent in Vero cells. Since the envelope proteins are the main determinants for binding to attachment and receptor molecules, receptor binding is still possible to be impacted by these two adaptations [30,31,32].

CHIKV entry is a versatile step, and depending on the cell type, various entry pathways, attachment factors, and receptors have been described. MXRA8 is, so far, the only CHIKV receptor for which direct interaction with the CHIKV envelope has been shown, and for which the interaction residues have been identified [31,32,33,34,35]. The two adapted residues found here are not positioned within the MXRA8 binding region, although they are located in close proximity to this region (Supplementary Figure S5). It was not expected to find adaptations that increase MXRA8 binding in A549 cells since it has been described that A549 cells do not express MXRA8 [33]. A549 cells, however, do express other described alphavirus entry factors like CD147 [19]. Interestingly, residue H232 has previously been found to be located within the target epitope of various neutralizing antibodies (CHK-9, CHK-152, CHK163, and m242), which block infection by preventing CHIKV binding and attachment and/or fusion [36,37,38]. Also, residue T196, although not directly targeted by neutralizing antibodies, is positioned in a region with multiple binding sites for neutralizing antibodies [38]. Although the acquired mutations probably do not affect MXRA8 binding since attachment is similarly improved in A549 and Vero cells, it is possible that they facilitate better binding to other known or unknown receptor and/or attachment factors. The observation that these acquired mutations have an opposite effect on CHIKV infection in Vero cells, not related to a change in viral attachment, could suggest that attachment and/or entry factors in A549 and Vero cells are expressed differently and/or targeted or used differently by the virus.

Of the four acquired amino acid substitutions, nsP1-M314V arose first and contributes most to the viral phenotype in A549 cells. nsP1 assists in the capping of the viral RNA, and as a membrane anchor, it functions to locate the replication complexes to the host cellular membranes [39]. In addition, nsP1 downregulates the expression of tetherin, a virus restriction factor retaining virus particles on the cell surface [40]. Three domains have been described for nsP1, the methyltransferase and guanylyltransferase domain, the membrane binding domain, and the C-terminal D3 domain, which is suggested to be important for nsP1 oligomerization [41,42]. It is in the latter domain that the acquired mutation can be found. In nature, the mutation has been described four times in different East/Central/South African (ECSA) CHIKV strains (SK2479_18, SK2461_18, SK2597_19, and SK2614_19) isolated in Thailand in 2018-2019. Also, the HB78 strain isolated in 1978 in the Central African Republic, but passaged seven times in cell culture, harbors this mutation [43]. Interestingly, the nsP1-M314V adaptive mutation to A549 cells not only allowed a better replication in A549 cells but also significantly increased CHIKV replication in Vero cells. Preliminary experiments performed on mosquito C6/36 cells indicate that also in these cells, the nsP1-M314V mutation significantly increased CHIKV replication (data not shown). Since Vero and C6/36 cells already support efficient CHIKV replication, there is no genetic pressure or need for these adaptive mutations to arise in these cells. This suggests that the adaptive mutations preferentially arise in a cell with a higher genetic barrier (A549) than in a cell (Vero or C6/36) with a lower genetic barrier (Vero or C6/36) for CHIKV replication.

The H687Y mutation found in nsP2 is located in the C-terminal protease domain, essential for the proteolytic processing of the non-structural polyprotein during formation of the replicase complex. Apart from its enzymatic functions, nsP2 is central in suppressing cellular transcriptional and antiviral responses [39]. So far, H687Y has been found only once in an Asian CHIKV strain isolated in Thailand in 1995 (SV0444-95) [43]; no other primary strain has carried this mutation. Interestingly, ectopic peptide insertions at this site in SINV led to a diminished suppression of innate immune signaling [44]. This indicates that the nsP2-H687Y mutation could play a role in suppressing innate immune signaling, thereby promoting viral replication. On its own, the mutation had only minimal effects on CHIKV replication in A549 cells, and no additional effect was observed when combined with the mutation in nsP1. Sequence analysis showed that the appearance of the nsP2 mutation probably followed the nsP1 mutation (Supplementary Figures S2 and S3). The four non-structural proteins interact to form the replication complex that synthesizes viral RNA [39]. The interaction between nsP1 and nsP2 in this replication complex has been studied to some degree, and the main interacting domains responsible for this interaction are residues 1–95 of nsP2 and 170–288 of nsP1 [42,45]. Since both acquired nsP mutations lie outside these interaction domains, this suggests that the nsP mutations probably were not acquired because of a change in the nsP1-nsP2 interaction efficiency [42,45].

With the trans-replicase assay, we investigated the role of the nsP mutations on the efficiency of viral genomic replication. However, no significant increase in RNA replicase activity was noted. Given that the relative amounts of replicase proteins on the one hand, and the viral genomic template on the other, might be different in the trans-replicase assay compared to the infectious virus, the TRA could conceivably underestimate a role for the nsP mutations in viral genomic replication. While mutations that interfere with efficient viral replication in infectious viruses that do not impede TRA activity have been described (mutations found in crucial viral regulatory RNA elements), neither of the nsP mutations identified here were found in these regions.

Therefore, we also consider that the nsP1 mutation, shown to be critical for increased viral replication, plays a role outside the viral replication complex. This indicates the presence of a so-far unknown restriction mechanism for alphaviral replication in A549 cells. The antiviral factor tetherin is a potential candidate as it is known that nsP1 downregulates its expression [40]. The nsP1-M314V mutation may increase tetherin downregulation, allowing a better release of viral particles at the cell membrane. However, further studies will be necessary to understand how the acquired mutations help CHIKV overcome the natural occurring block in CHIKV replication in A549 cells.

In conclusion, after serial passaging of CHIKV on restrictive A549 cells, four mutations were rapidly acquired to enable CHIKV to replicate in A549 cells. Of these mutations, two were found in the envelope proteins and two in the non-structural proteins. While the mutations in the non-structural proteins also contributed to an increased CHIKV replication in permissive Vero cells, the mutations in the structural proteins impeded CHIKV replication in Vero cells. Previous adaptive mutations in CHIKV E1 have been reported that facilitated transmission by *Aedes albopictus* mosquitoes. Our findings re-emphasize the genomic plasticity of alphaviruses to overcome restriction barriers at the cellular level.

## Figures and Tables

**Table 1:** Primers for CHIKV genome amplification.

5′ Primer F	3′ Primer R	Fragment
ATGGCTGCGTGAGACACACGTAGC	TTGCTTCATCCAGCTTAGGTGGG	19—5816
AGCGACTGGTCCACGTGCT	GAAATATTAAAAACAAAATAACATCTCCTACGTCCCTGT	5642—11829

## Data Availability

Not applicable.

## Supplementary Materials

The following supporting information can be downloaded at: https://www.mdpi.com/article/10.3390/v14020282/s1, Figure S1: Sequence analysis of E2, Figure S2: Sequence analysis of nsP2, Figure S3: Sequence analysis of nsP1, Figure S4: Sequence analysis of E1, Figure S5: Structure of the Chikungunya Virus (CHIKV) Envelope Proteins, Data S1: contig sequences CHIKV A549 adap.
