# Supplementary material for: Chikungunya Virus’ High Genomic Plasticity Enables Rapid Adaptation to Restrictive A549 Cells
Source: Viruses. 2022 Jan 28;14(2):282. doi: 10.3390/v14020282 (PMC8879786; doi:10.3390/v14020282)
Supplement: Supplementary file 1 [file viruses-14-00282-s001.zip › Supplementary Materials CHIKV A549 LDC.pdf]

Supplementary Materials:

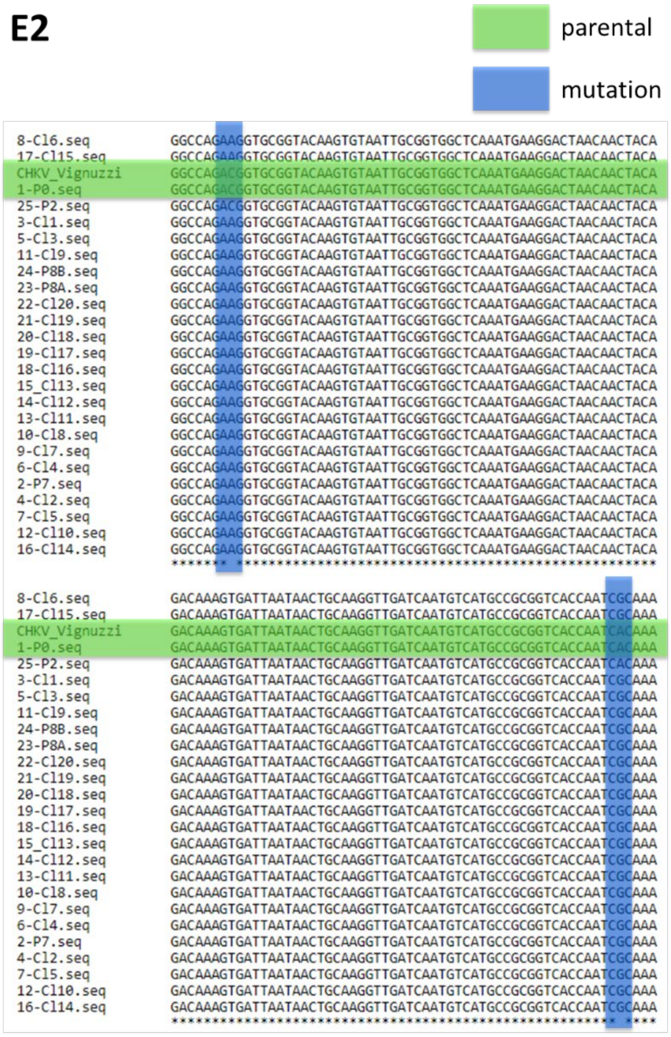

Figure S1. Sequence analysis of E2. Passages P0, P2, P7 and P8 were sequenced together with 20 separately plaque purified monoclonal viruses from P8.

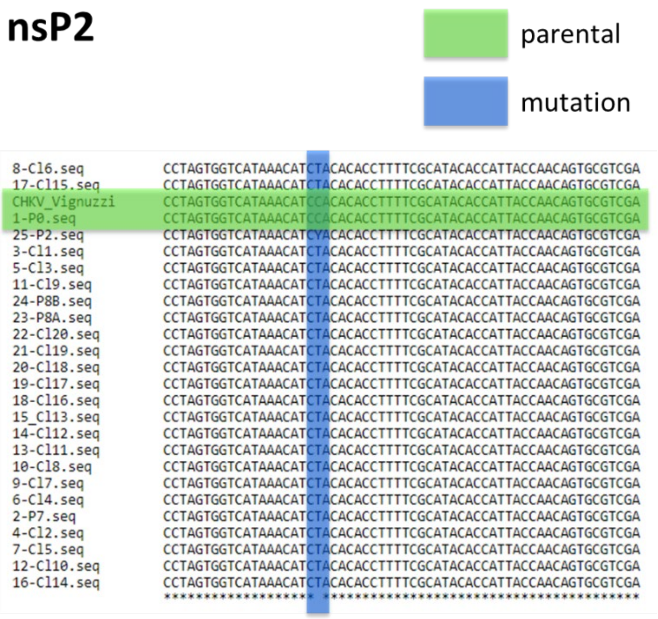

Figure S2. Sequence analysis of nsP2. Passages P0, P2, P7 and P8 were sequenced together with 20 separately plaque purified monoclonal viruses from P8.

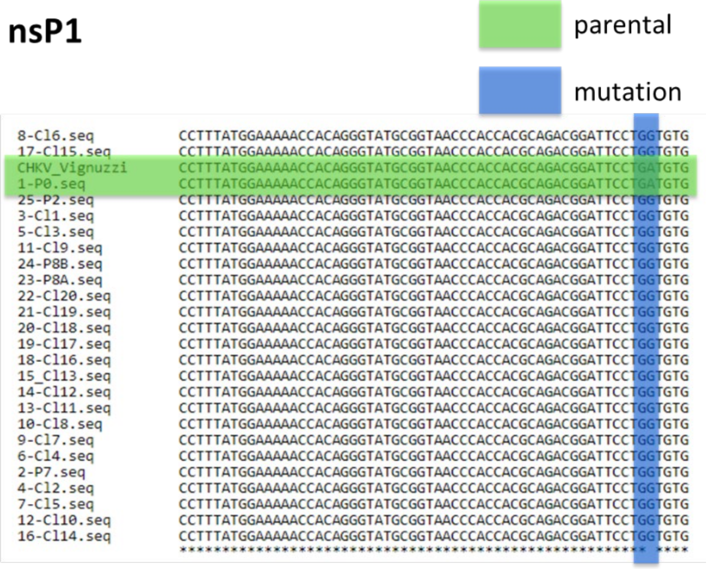

Figure S3. Sequence analysis of nsP1. Passages P0, P2, P7 and P8 were sequenced together with 20 separately plaque purified monoclonal viruses from P8.

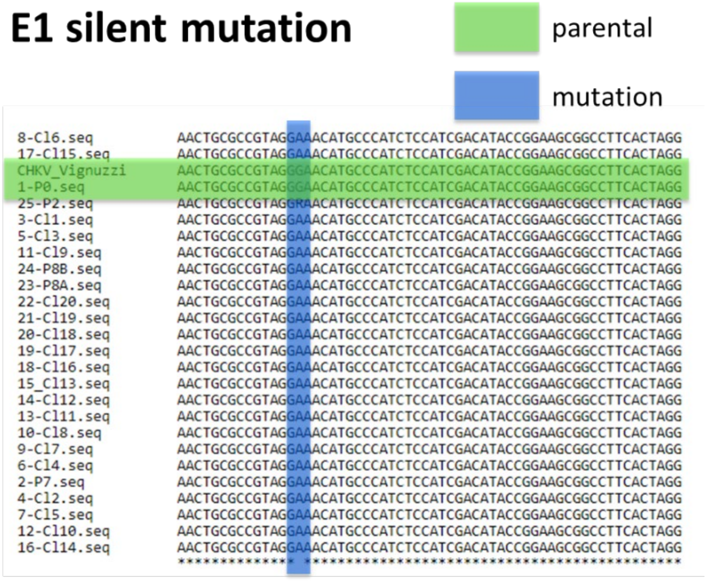

Figure S4. Sequence analysis of E1. Passages P0, P2, P7 and P8 were sequenced together with 20 separately plaque purified monoclonal viruses from P8.

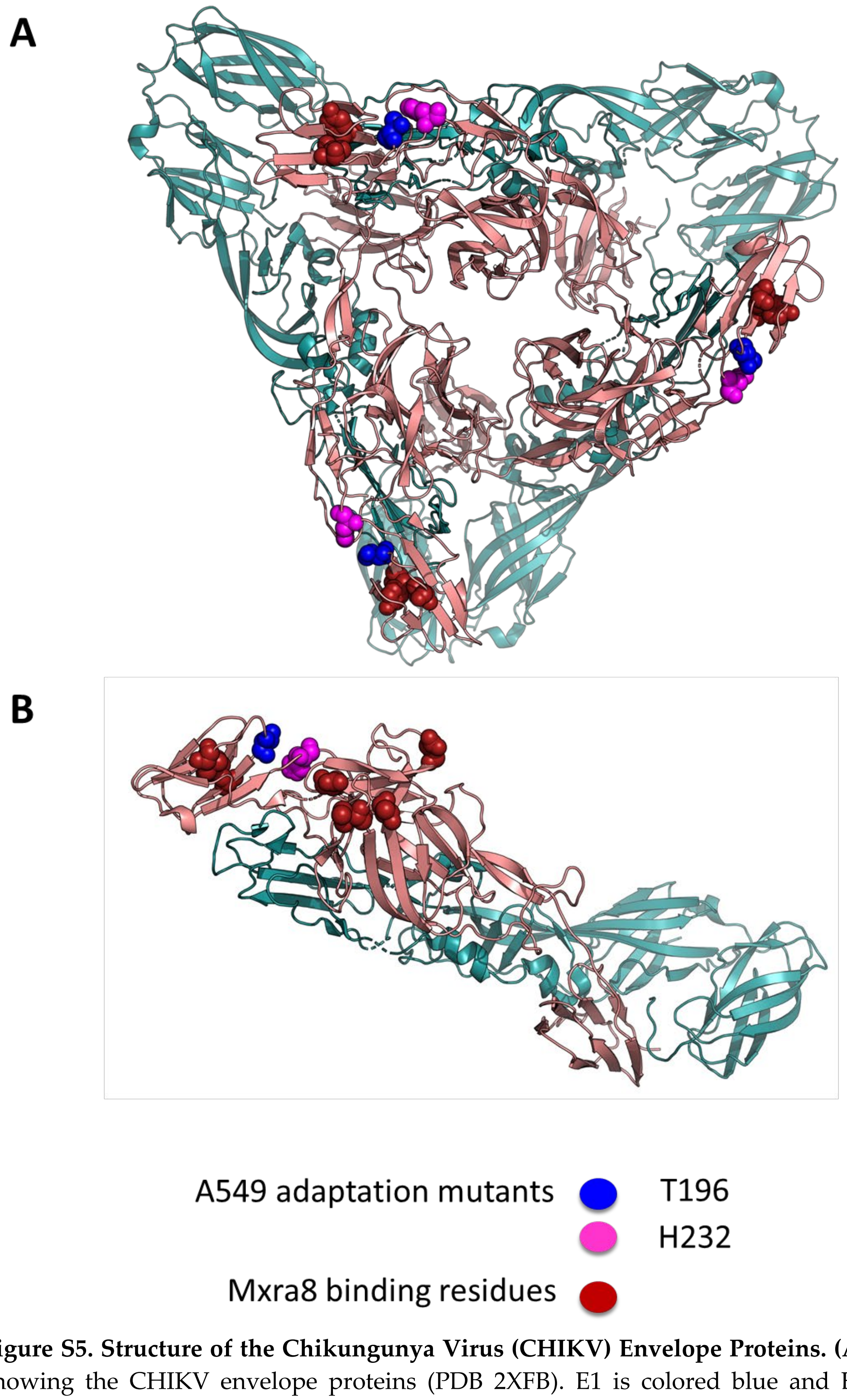

Figure S5. Structure of the Chikungunya Virus (CHIKV) Envelope Proteins. (A,B) Ribbon diagram showing the CHIKV envelope proteins (PDB 2XFB). E1 is colored blue and E2 is pink. Adaptive mutations are shown as blue (T196) and purple (H232) spheres, MXRA8 binding residues are red spheres. **(A)** Top view of E1-E2 trimer. **(B)** E1-E2 dimer.
